# Intestinal stem cells: guardians of homeostasis in health and aging amid environmental challenges

**DOI:** 10.1038/s12276-024-01179-1

**Published:** 2024-03-01

**Authors:** Jiahn Choi, Leonard H. Augenlicht

**Affiliations:** 1 Department of Cell Biology, Albert Einstein College of Medicine, Bronx, NY USA; 2 Department of Medicine, Albert Einstein College of Medicine, Bronx, NY USA

**Keywords:** Intestinal stem cells, Ageing

## Abstract

The intestinal epithelium is the first line of defense and acts as an interface between the vast microbial world within the gastrointestinal tract and the body’s internal milieu. The intestinal epithelium not only facilitates nutrient absorption but also plays a key role in defending against pathogens and regulating the immune system. Central to maintaining a healthy epithelium are intestinal stem cells (ISCs), which are essential for replenishing the intestinal epithelium throughout an individual’s lifespan. Recent research has unveiled the intricate interplay between ISCs and their niche, which includes various cell types, extracellular components, and signaling molecules. In this review, we delve into the most recent advances in ISC research, with a focus on the roles of ISCs in maintaining mucosal homeostasis and how ISC functionality is influenced by the niche environment. In this review, we explored the regulatory mechanisms that govern ISC behavior, emphasizing the dynamic adaptability of the intestinal epithelium in the face of various challenges. Understanding the intricate regulation of ISCs and the impact of aging and environmental factors is crucial for advancing our knowledge and developing translational approaches. Future studies should investigate the interactive effects of different risk factors on intestinal function and develop strategies for improving the regenerative capacity of the gut.

## Aging and environment: their impact on the gut’s regenerative capacity

This study, led by Jiahn Choi and Leonard H. Augenlicht, explores intestinal stem cells. The researchers carried out a review, studying how ISCs work and how factors like aging and diet influence them. They used various methods, including gene expression profiling and single-cell RNA sequencing, to comprehend how ISCs maintain the gut and how this alters with age or different diets. The researchers discovered that aging and diet greatly impact ISCs and the cells that assist them. For instance, aging can lessen the ability of ISCs to renew the gut lining, while certain diets can either damage or enhance ISC function. These findings propose that maintaining a healthy diet could be crucial to preserving gut health, especially as we age. The study progresses our understanding of the gut’s maintenance system and suggests that lifestyle choices are significant for gut health.

This summary was initially drafted using artificial intelligence, then revised and fact-checked by the author.

## Introduction

The gastrointestinal tract is an extraordinary biological system that serves as a complex interface between the vast microbial world within the digestive system and the internal milieu of the body^1^. At the forefront of this intricate ecosystem lies the intestinal epithelium, which is a single layer of specialized cells that lines the inner surface of the gut^2,3^. This remarkable tissue is responsible for vital functions such as nutrient absorption, the maintenance of a robust barrier against pathogens, and the regulation of immune responses within the digestive system. Intestinal stem cells (ISCs) are central to the continuous regeneration of this essential tissue^3–5^.

ISCs play a pivotal role in maintaining the structural integrity and functional capacity of the intestinal epithelium. These remarkable cells reside within small pockets, known as crypts, and are entrusted with the extraordinary responsibility of ensuring the continual renewal and replenishment of the intestinal lining throughout an individual’s lifetime^6^. ISCs exhibit remarkable plasticity and are capable of self-renewal and differentiation into diverse cell types that are required for normal intestinal function^7^. Through a precisely orchestrated process, ISCs proliferate and generate into specialized cell lineages, including absorptive enterocytes, mucus-secreting goblet cells, hormone-producing enteroendocrine cells, antimicrobial peptide-releasing Paneth cells, etc.^8^. This careful balance of self-renewal and differentiation is orchestrated by intricate signaling pathways, such as the Wnt pathway, which is crucial for coordinating the seamless regeneration of the intestinal epithelium. In this review, we focus primarily on recent advances in understanding the regulatory function of ISCs in homeostasis and how it is influenced by various risk factors for disease, particularly tumorigenesis and inflammation.

## Regulation of canonical ISC functions

**Fig. 1:**
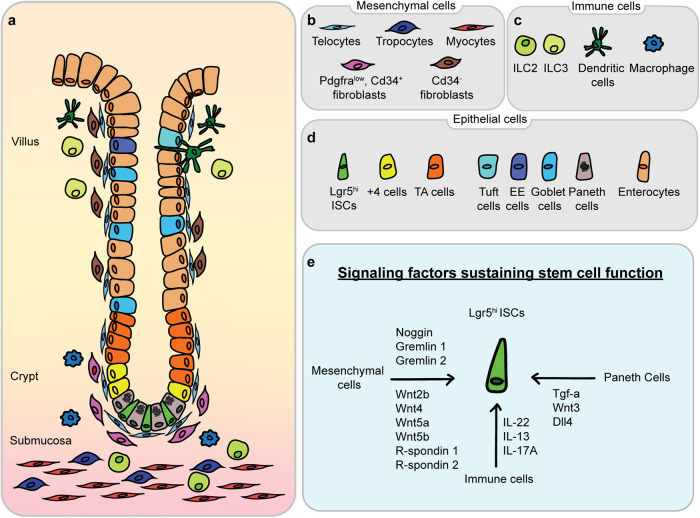
Overview of the intestinal stem cell niche and associated signaling pathways. **a** Schematic representation of the intestinal crypt-villus axis and its surrounding niche. Intestinal stem cells are found at the base of the crypt; neighboring niche cells include Paneth cells, telocytes, Pdgfra^low^, and Cd34+ fibroblasts. ISCs receive paracrine growth factors from the niche cells that support stem cell functions. Cd34- fibroblasts are mainly found in the villus. Different types of immune cells are distributed along the crypt-villus axis. **b**–**d** Multiple cell types in the intestine were found in the intestinal mesenchyme (**b**), immune cells (**c**), and epithelium (**d**). **e** Signaling factors sustaining Lgr5^hi^ ISC functions; the interaction between Lgr5^hi^ ISCs and various niche components is shown.

Leucine-rich repeat-containing G protein-coupled receptor 5 (Lgr5) is a receptor for R-spondin that promotes Wnt signaling by stabilizing β-catenin^9^. The intricate balance between the self-renewal and differentiation of Lgr5^hi^ ISCs plays a vital role in maintaining the continuous replenishment of the intestinal epithelium, which allows for the preservation of its structural and functional integrity. Originally identified as a marker of actively cycling cells located at the base of crypts^8^, Lgr5 has become a widely utilized stem cell marker for tracking and isolating actively cycling stem cells in the intestine and other organs^3^. The regulation of ISCs and their behavior are influenced by many factors, both intrinsic and extrinsic. Disruptions in the regulation of Lgr5^hi^ ISCs can lead to perturbations in mucosal homeostasis, potentially resulting in gastrointestinal diseases, including inflammation, inflammatory bowel disease, and tumorigenesis. The complex interplay between cellular composition and paracrine signaling molecules, which shape the intestinal epithelium and its supportive niche, is depicted in Fig. 1.

### Signaling pathways that regulate the function of ISCs

Multiple developmental signaling pathways, including the Wnt, Notch, BMP, and Hedgehog pathways, play pivotal roles in governing the fate and function of ISCs^10–13^. The Wnt pathway, in particular, is a major contributor to the maintenance and function of ISCs. Transcriptional regulators such as Lgr5 and Ascl2 are critical for sustaining the ISC pool and controlling the proliferative potential of ISCs^14^. Recent research has highlighted the importance of TRIM27 in maintaining gut homeostasis. TRIM27 competes with Axin for binding to the conserved Armadillo (ARM) repeat domain of β-catenin, thus disrupting the Axin-β-catenin interaction. This interference leads to the stabilization of β-catenin and its subsequent translocation from the cytoplasm to the nucleus, thereby activating the Wnt/β-catenin signaling pathway. Such activation is fundamental for the self-renewal of Lgr5^hi^ ISCs and the production of epithelial lineages and is crucial for maintaining the integrity of the intestinal epithelial barrier and gut homeostasis^15^. Vitamin D receptor (Vdr) expression is also important for maintaining the function of ISCs. The Vdr gene was identified as one of the 30 core components of the stem cell gene signature of Lgr5^hi^ ISCs. The robust expression of Vdr in Lgr5^hi^ cells is immediately downregulated in daughter cells, coinciding with their loss of function as stem cells^16^. The deletion of Vdr in Lgr5^hi^ ISCs inhibited lineage tracing, thereby confirming the necessity of Vdr expression for normal ISC function^17^. In addition, activation or upregulation of Vdr expression attenuates radiation-induced intestinal damage in mice and promotes the repair of epithelial damage in intestinal stem cells; thus, these results further establish the key role of Vdr signaling in the function of Lgr5^hi^ ISCs^18^.

### Intestinal stem cell niche

Within the complex microenvironment of the gastrointestinal tract lies a vital determinant of mucosal homeostasis — the stem cell niche. This specialized microenvironment delivers essential signals and cues critical for the maintenance, regulation, and functionality of ISCs^19^. A fundamental component of the ISC niche is the Paneth cell, which interacts with Lgr5^hi^ ISCs at the base of crypts. Moreover, Paneth cells secrete growth factors, antimicrobial peptides, and Wnt ligands, thereby contributing to mucosal immune defense and sustaining stem cell function. The role of these cells as niche cells encompasses the release of important growth factors, including Egf, Tgf-a, Wnt3, and the Notch ligand Dll4, each of which activates critical signals that are necessary for stem cell maintenance and function^20^.

Mesenchymal cells also form a significant part of the niche, with distinct subsets including pericryptal myofibroblasts, fibroblasts, pericytes, endothelial cells, immune cells, neural cells, and smooth muscle cells^12,21,22^. Single-cell RNA sequencing (scRNAseq) has revealed a diverse population of intestinal mesenchymal cells characterized by the expression of marker genes, such as Gli1, Pdgfra, and Foxl1^23–25^. Twist2 stromal cells were recently identified as a niche subpopulation for canonical ISCs that maintain homeostasis through the secretion of Wnt ligands^26^. Further studies utilizing scRNAseq revealed new stromal cell types, including MAP3K2-regulated stromal cells and RSPO3^+^GREM1^+^ fibroblasts, which were sources of RSPO1 and RSPO3^27,28^. The regulation of ISC potential is also influenced by signals from lymphatic cells. In addition to the newly identified protein REELIN, Wnt2 and R-spondin3, which are both Wnt signaling factors, directly regulate the regenerative capacity of ISCs as crypt lymphatic signals^29^.

Immune cells predominantly reside in the lamina propria just beneath crypts^30^. Together with other stromal cells, such as fibroblasts and endothelial cells, immune cells constitute the ISC microenvironment and orchestrate the complicated processes of ISC self-renewal and differentiation^31^. Immune cells regulate ISCs through the production of cytokines and other stimulatory factors, thereby influencing the integrity of the intestinal barrier, which plays an important role in the pathogenesis of intestinal and systemic diseases^32^. For example, IL-22 secreted by type 3 innate lymphoid cells (ILC3s) can promote ISC-mediated epithelial regeneration after intestinal damage and drive ISC self-renewal^33^. In addition, ILC2s can promote ISC self-renewal via the IL-13 pathway^34^. Cytokines secreted by immune cells also guide the differentiation of Lgr5^hi^ ISCs. A recent study showed that IL-17A regulates the expression of Atoh1, a key component in lineage specification in the Notch pathway in Lgr5^hi^ ISCs^35^, thus inducing differentiation toward secretory cell types^36^.

## Extrinsic factors that disrupt mucosal homeostasis

**Fig. 2:**
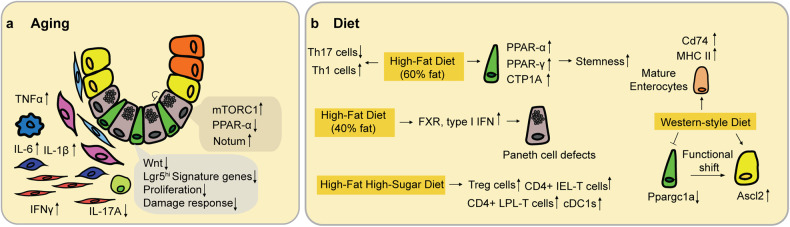
The impact of extrinsic factors that perturb intestinal homeostasis. **a** Summary of how aging alters ISC function, as well as the ISC niche. **b** Summary of how diet influences ISC function, as well as the ISC niche. The nutritional composition and the relative levels of nutrients in mouse diets significantly change the adaptive responses of the intestinal epithelium.

Homeostasis is a continuous process of tissue/organ modification and adaptation^37^. In the intestine, this process alters mucosal structure, function, and regenerative capabilities through reprogramming of ISCs, progenitor cells, and their lineages. Perturbations in the adaptive response of the intestinal epithelium cause maladaptation to extrinsic environmental factors, further aggravating mucosal homeostasis, as depicted in Fig. 2.

### Aging

Aging is a natural and inevitable process that is characterized by progressive physiological and functional decline across multiple organ systems, and the gastrointestinal tract is no exception. This decline is critical for understanding the increase in vulnerability to gastrointestinal disorders such as inflammation and tumorigenesis.

A key age-related alteration is the reduced regenerative capacity of the intestinal epithelium^38^. ISCs, which are critical for continual replenishment of the epithelium, exhibit a marked reduction in their proliferative potential and self-renewal ability with age^39,40^. Investigations of aging mice have shown that they have lower survival rates and their intestinal crypts undergo significantly less cell division than normal crypts^41^. Gene expression profiling revealed aging-associated changes in mRNAs associated with the cell cycle, oxidative stress and apoptosis^39^. Using scRNAseq technology, a recent study demonstrated that a functional decline in ISCs leads to a delay in progenitor cell maturation, potentially leading to the overall impairment of regenerative capacity^42^. This results in a diminished capacity to replace damaged or dying cells, ultimately compromising the regenerative response to injury or stress within the mucosal lining and reducing the overall efficiency of maintaining mucosal function and integrity. Additionally, the altered behavior and functionality of ISC niches, including changes in the secretory profile of Paneth cells and the composition of the extracellular matrix, contribute to the perturbation of mucosal homeostasis during aging. The functional decline in ISCs was caused by a decrease in Wnt signaling due to the production of Notum, an extracellular Wnt inhibitor, in aged Paneth cells^43^. The mechanism involved the high activity of mammalian target of rapamycin complex 1 (mTORC1) in aged Paneth cells, which inhibited the activity of peroxisome proliferator activated receptor α (PPAR-α), and decreased PPAR-α activity, which increased Notum expression^43^. Another study employed bulk genome-wide chromatin accessibility and transcriptome analysis combined with scRNAseq and revealed that promoters of polycomb target genes become differentially accessible in aged ISCs, resulting in increased expression of genes involved in enteroendocrine cell specification and biased differentiation^44^. In addition, the ability to repair cellular organization in the intestinal crypt is also diminished in aging mice^45^. This study revealed the unique ‘soccer ball-like’ mosaic pattern formed by Lgr5^hi^ ISCs surrounding Paneth cells, which is rapidly restored upon disruption by the rearrangement of cellular organization. This process accompanies the active clearance of cellular debris, depending on orchestrated movement within the stem cell niche. Importantly, the aged ISC niche exhibited less repair due to diminished motility, resulting in delays in post-damage recovery^45^.

Aging has a multifactorial impact on the immune system in the intestine. The proper immune functions of the tissue are impaired, but the number of immune cells is also elevated, leading to low-level chronic inflammation. This process has been termed inflammaging, a hallmark of aging that significantly impacts mucosal homeostasis. Intestinal levels of proinflammatory cytokines, in particular TNFα, IL-1β, IFNγ and IL-6, tend to increase with age^5,46^. The sustained inflammatory state disrupts the delicate balance of immune responses within the gastrointestinal tract, thereby altering the gut microbiome composition, affecting nutrient absorption, and impairing the barrier function of the intestinal epithelium. Ultimately, the accumulation of age-associated changes contributes to an altered mucosal microenvironment, which disrupts the functional harmony of the intestinal epithelium and predisposes older individuals to gastrointestinal ailments^47^. Studies have also shown that the amount of CD8+ T cells in intestinal lamina propria mononuclear cells increases in elderly individuals, with a striking increase in CD45R- memory T cells with age^48^. The profile of highly proliferative and activated T cells in the aged gut reflects the skewing of lymphocytes from a naïve state to a memory and/or effector state during lifetime antigen exposure^49^. Despite extensive investigations in this area, further work is necessary to determine the impact of aging on intestinal immunity, and additional studies need to investigate how alterations in immune cell populations and the phenomenon of inflammaging influence the function of ISCs and progenitor cells.

### Diet

Dietary patterns and nutritional components are among the most influential environmental factors that affect mucosal homeostasis. A diet rich in processed foods, high in sugars and unhealthy fats, and low in fiber has been linked to inflammation, altered gut microbiota composition, and compromised barrier function^50^. Conversely, fiber-rich diets with ample antioxidants and essential nutrients support a healthy gastrointestinal tract by promoting microbial diversity, enhancing immune function, and facilitating proper digestion and absorption^51^. One challenge is pinpointing specific changes to individual nutrients, as they often substantially interact to influence cellular reprogramming in the intestine and are contingent on the presence and levels of other nutrients. This complexity is not surprising, given that even potent oncogenes and tumor suppressor genes can exhibit varying or null effects across different tissues, depending on their distinct transcriptional programs. Despite these complexities, focused research has yielded significant insights. A recent study demonstrated that a high-fat diet comprising 60% fat enhances the self-renewal capacity of Lgr5^hi^ ISCs through PPARα/δ-dependent activation of downstream pathways, including β-catenin and fatty acid oxidation^52^. Extended exposure to the same high-fat diet suppresses the expression of major histocompatibility complex (MHC) class II molecules in ISCs^53^. However, the adaptive response to diet varies depending on the diet composition and relative nutrient levels. In another study, a Western-style diet in which a number of nutrients were adjusted was linked to increased risk^54^, which induced impairment of Lgr5^hi^ ISCs via epigenetic downregulation of the expression of *Ppargc1a*, a master regulator of mitochondrial biogenesis. In this study, alternate stem cells that were Bmi1+ Ascl2^hi^ were recruited to compensate for the functional impairment of Lgr5^hi^ ISCs, which subsequently led to the remodeling of the lineages^55^.

Nutrient exposure also has a tremendous impact on the development of abnormal phenotypes in Paneth cells. Mice fed a Western-style diet adjusted to reflect nutrient levels typically found in humans with a higher risk of tumorigenesis exhibited ectopic expression of Paneth cell markers throughout the intestinal mucosa^56^. A high-fat diet with a fat content of 40% was used to induce Paneth cell defects through two key pathways within the intestinal epithelium—the farnesoid X receptor (FXR) pathway and the type I interferon (IFN) pathway. Both pathways are needed to induce alterations, as inhibition of either FXR or type I IFN signaling prevents high-fat diet-induced Paneth cell defects^57^.

Dietary exposure also affects the intestinal immune system. Multiple studies using dietary conditions that reflect an elevated risk for tumorigenesis have shown low-grade chronic inflammation. Extended exposure to a Western-style diet increased MHC II complex expression in mature enterocytes and increased the number of tissue-resident immune cells^55^. This condition has been documented in various etiologies of human inflammatory bowel disease and is also protumorigenic^58,59^. Feeding a high-fat high-sugar (HFHS) diet dramatically increased the proportion of regulatory T (Treg) cells, as well as that of CD4+ intraepithelial lymphoid-T cells (IEL-T cells), CD4+ lamina propria lymphoid-T cells, and conventional type 1 dendritic cells (cDC1s), which are critical for Treg induction. Moreover, memory-like CD8αα + IEL-T cells and memory-like CD8αβ + IEL-T cells accumulate in the intestines of mice fed a HFHS diet; these cells express high levels of XCL1 and are likely involved in the recruitment of cDC1s via the XCL1–XCR1 signaling axis in response to the diet^60^. Obesity has also been linked to a nonspecific, low-grade inflammatory state in the intestine accompanied by the activation of proinflammatory signaling pathways, such as the NF-κB pathway, and increased expression of cytokines, including IL-1β, TNFα, and IL-12p40^61,62^. A high-fat diet also shifts the immune cell balance in the small intestinal lamina propria by elevating the level of IFNγ-producing Th1 cells and CD8 + T cells while reducing that of immunosuppressive Treg cells^63^. Additionally, feeding a high-fat diet also reduced the abundance of IgA+ B cells in the gut of mice, especially in the small intestine and lamina propria^64^.

### Microbiome

The gut microbiome, which is a complex consortium of microorganisms within the gastrointestinal tract, plays a pivotal role in shaping mucosal homeostasis. Importantly, the intestinal microbiome has a bidirectional impact on gut homeostasis and pathogenesis. In aging individuals, the important role of the gut microbiota is highlighted by the notion that in centenarians, “longevity adaptation” is characterized by enrichment in health-associated gut microbes^65,66^.

The majority of adults over 65 years of age exhibit reduced microbiota diversity compared to younger adults, along with greater interindividual variation in microbiota composition^67^. This may be reflected in the observation that mice from the same colony and subjected to the same environmental conditions (i.e., housing, diet, temperature, humidity, etc.) exhibited an age-dependent drift in their microbiota profile^68,69^, likely indicating that normal mechanisms that help maintain the balance of complex ecosystems deteriorate with age. Moreover, in a murine model, intrinsic, environment-independent aging itself drove alterations in the gut microbiome composition^70^. The senescence-related remodeling of microorganisms mediates an increase in chronic inflammation, which is strongly correlated with microbial metabolites and induced immune responses^68,71^. Disruptions of the gut microbiota composition, often triggered by antibiotic use, microbial infections, or excessive hygiene, can lead to dysbiosis, inflammation, and compromised mucosal barrier integrity. A recent study showed that defects in Paneth cells induced dysbiosis, resulting in the activation of tuft cells and further triggering type 2 immune responses^72^. Furthermore, lactate from lactic acid-producing bacteria plays a pivotal role in promoting ISC proliferation and epithelial development^73^. Nevertheless, further research is required to comprehensively understand the mechanisms by which microbial metabolites influence the function of ISCs, reprogramming of the epithelial mucosa, and the onset of pathogenesis.

## Conclusion

Epithelial regeneration serves as the linchpin for preserving intestinal barrier function and facilitating efficient nutrient absorption. A decrease in regenerative capacity is a defining hallmark of aging in the intestine; thus, there has been a push for extensive research into how regeneration is stimulated and regulated. To advance our understanding and develop translational approaches for addressing age-related intestinal decline, the complexity of the gut ecosystem and the intricacies of stem cell regulation need to be addressed. While significant progress has been made, there are notable gaps, especially regarding the comprehensive assessment of multiple risk factors in the context of the complex interplay of aging, diet, the microbiome, and other environmental variables. While this research is challenging, it is essential for developing new approaches to promote gastrointestinal function and health across the lifespan.
